# Use of Robots in Critical Care: Systematic Review

**DOI:** 10.2196/33380

**Published:** 2022-05-16

**Authors:** Rachel Teng, Yichen Ding, Kay Choong See

**Affiliations:** 1 Yong Loo Lin School of Medicine National University of Singapore Singapore Singapore; 2 Division of Respiratory & Critical Care Medicine Department of Medicine National University Hospital Singapore Singapore

**Keywords:** COVID-19, intensive care, high dependency, telepresence, intubation

## Abstract

**Background:**

The recent focus on the critical setting, especially with the COVID-19 pandemic, has highlighted the need for minimizing contact-based care and increasing robotic use. Robotics is a rising field in the context of health care, and we sought to evaluate the use of robots in critical care settings.

**Objective:**

Although robotic presence is prevalent in the surgical setting, its role in critical care has not been well established. We aimed to examine the uses and limitations of robots for patients who are critically ill.

**Methods:**

This systematic review was performed according to the PRISMA (Preferred Reporting Items for Systematic Reviews and Meta-Analyses) guidelines. MEDLINE, Embase, IEEE Xplore, and ACM Library were searched from their inception to December 23, 2021. Included studies involved patients requiring critical care, both in intensive care units or high-dependency units, or settings that required critical care procedures (eg, intubation and cardiopulmonary resuscitation). Randomized trials and observational studies were included.

**Results:**

A total of 33 studies were included. The greatest application of robots in the intensive care unit was in the field of telepresence, whereby robots proved advantageous in providing a reduced response time, earlier intervention, and lower mortality rates. Challenges of telepresence included regulatory and financial barriers. In therapy and stroke rehabilitation, robots achieved superior clinical outcomes safely. Robotic use in patient evaluation and assessment was mainly through ultrasound evaluation, obtaining satisfactory to superior results with the added benefits of remote assessment, time savings, and increased efficiency. Robots in drug dispensing and delivery increased efficiency and generated cost savings. All the robots had technological limitations and hidden costs.

**Conclusions:**

Overall, our results show that robotic use in critical care settings is a beneficial, effective, and well-received intervention that delivers significant benefits to patients, staff, and hospitals. Looking ahead, it is necessary to form strong ethical and legislative frameworks and overcome various regulatory and financial barriers.

**Trial Registration:**

PROSPERO International Prospective Register of Systematic Reviews CRD42021234162; https://www.crd.york.ac.uk/prospero/display_record.php?RecordID=234162

## Introduction

Robotics is a rising field in the context of health care [1]. Although there has been a surge in the popularity of automated and semiautomated processes in robotic surgery, little research has been conducted on robotic use outside surgical settings. The recent focus on critical care settings, especially in light of the COVID-19 pandemic, with more patients requiring intensive care, monitoring, and treatment, has accentuated the importance of minimizing contact-based care while ensuring efficiency [2]. With regard to the perception and acceptance of robots by health care workers, the COVID-19 pandemic has certainly emphasized the need for more widespread robotic use.

However, there may be underlying concerns with regard to robot safety and job replacements. We hypothesize that, given the current robotic technology, the benefits of robots may be limited to replacing mundane tasks and that use is limited by logistic, ethical, and financial barriers. Therefore, we aimed to examine the benefits and limitations of robots and uncover any significant applications of robotic technology in the critical care setting.

To better evaluate the use of robots against conventional methods of care in critical care settings, we reviewed randomized controlled trials (RCTs) and observational studies. We hope to provide information that allows clinicians and policy makers to assess various areas affected by robotic use and find an appropriate role for robots within the intensive care setting. In addition, we hope that our findings can stimulate further development of robotic technology, including its combination with artificial intelligence (AI).

## Methods

### Search Strategy and Selection Criteria

The study has been registered with PROSPERO (International Prospective Register of Systematic Reviews; CRD42021234162) and was performed according to the PRISMA (Preferred Reporting Items for Systematic Reviews and Meta-Analyses) guidelines [3]. A total of 2 authors (RT and YD) independently and systematically searched PubMed, Embase, IEEE Xplore, and ACM Library for all relevant studies published from inception to December 23, 2021, using the *patient or population, intervention, comparison, and outcomes* search strategy [4]. Multimedia Appendix 1 provides the detailed search strategy (Tables S1-S3 in Multimedia Appendix 1). In addition, other studies were identified by scanning the reference lists of articles. No limits were applied for language. Disagreements were resolved with the senior author (KCS).

Robots are defined as any machine capable of performing a series of actions, either autonomously or with external guidance. Critical care is defined as the care of patients with severe illnesses requiring intensive care, monitoring, and treatment. Studies were included if they were RCTs and observational studies reporting robotic use on human participants in critical care settings (intensive care unit [ICU], burns unit, high-dependency unit, critical care, and neonatal ICU [NICU]) or during procedures required in critical care settings (intubation, ventilation, tracheostomy, cannulation, resuscitation, and dialysis). Articles were excluded if they had an irrelevant topic, wrong patient type (nonhuman participants), or wrong setting (surgical setting). Gray literature (preprint and conference abstracts) was excluded because of incomplete descriptions of the relevant areas.

## Results

### Study Selection

**Figure 1:**
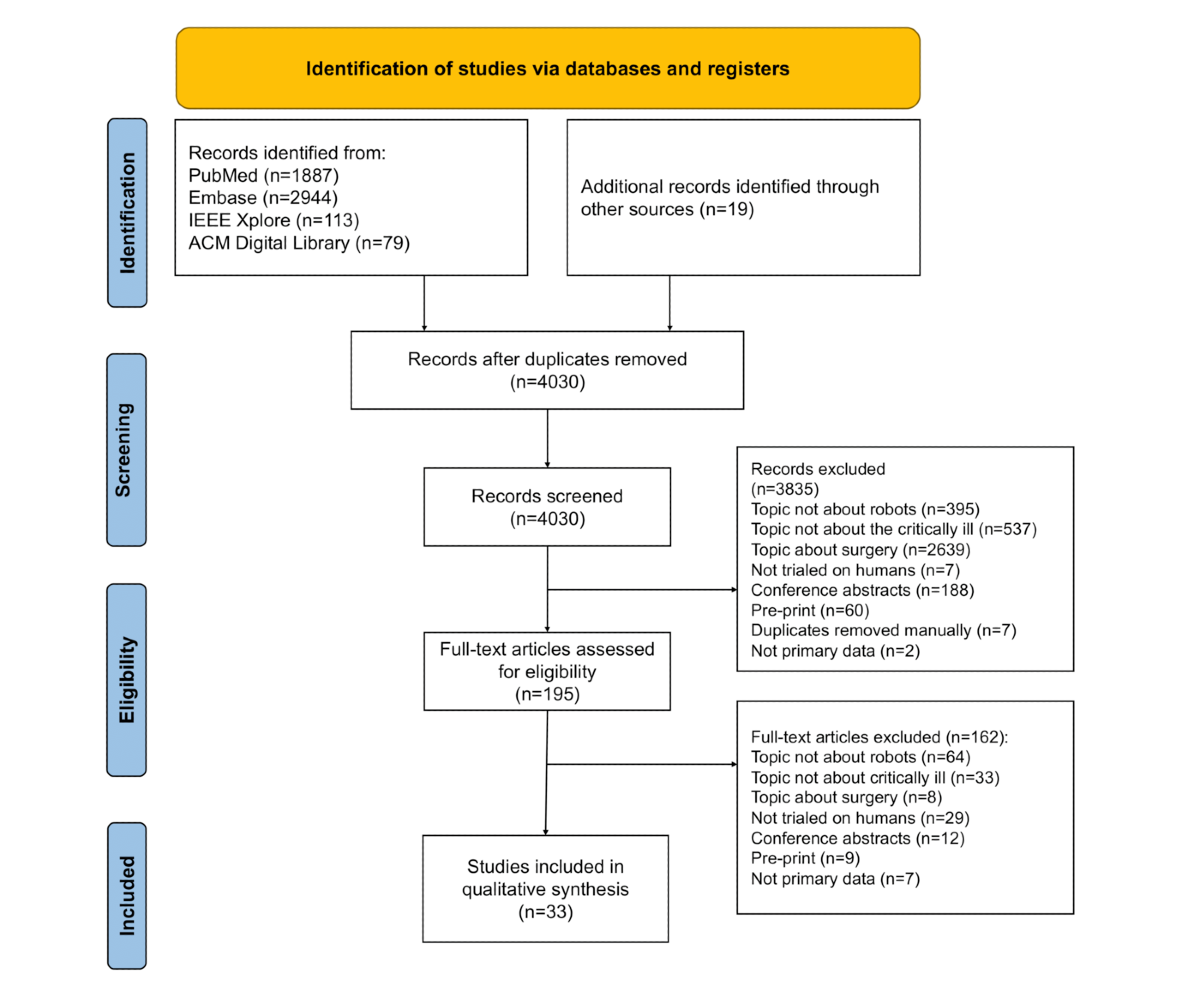
Study flow diagram.

PubMed, Embase, IEEE Xplore, ACM Digital Library, and reference list searches yielded a total of 5042 citations, of which 33 (0.65%) studies were identified for inclusion in the review (Figure 1).

### Data Extraction, Quality Assessment, and Data Synthesis

The extracted data included the benefits and limitations of robots. Included studies were independently assessed by 2 authors (RT and YD) for risk of bias using the Standard Quality Assessment Criteria (Tables 1 and 2) [5]. Each study was evaluated based on 14 criteria and scored according to the degree to which the criteria were met (yes, partial, or no). Items not applicable were marked as *N/A* and were excluded from the calculation of the summary score. Disagreements were resolved with the senior author (KCS). The Standard Quality Assessment Criteria suggests a cutoff point of 55% to 75% as an inclusion threshold. Of the 33 included studies, 27 (82%) attained a score of at least 65%. However, we did not exclude studies based on quality scores as this would arbitrarily limit data comprehensiveness.

With regard to data synthesis, given that study designs, participants, interventions, and reported outcomes were expected to vary across papers, we focused on the qualitative synthesis and did not conduct a meta-analysis. We have described the studies in terms of their results, applicability, and limitations.

**Table 1:** Risk of bias assessment of included studies using Standard Quality Assessment Criteria (study design and interventions).

Study	Study design					Interventions		
	Objective described	Evident and appropriate study design	Participant selection described and appropriate	Participant characteristics described	Random allocation described		Blinding of investigators reported	Blinding of participants reported
Adcock et al [6]	Yes	Yes	Yes	Yes	Yes		N/A^a^	N/A
Alnobani et al [7]	Yes	Yes	Yes	Partial	N/A		N/A	N/A
Amodeo et al [8]	Yes	Yes	Yes	Yes	N/A		N/A	N/A
Becevic et al [9]	Partial	Partial	No	Partial	N/A		N/A	N/A
Bettinelli et al [10]	Yes	Yes	Yes	Yes	Partial		N/A	N/A
Burke et al [11]	Partial	Partial	Partial	Partial	N/A		N/A	N/A
Duan et al [12]	Yes	Yes	Yes	Yes	N/A		N/A	N/A
Frazzitta et al [13]	Yes	Yes	Yes	Yes	Yes		N/A	N/A
Garingo et al [14]	Yes	Yes	Yes	Yes	N/A		N/A	N/A
Garingo et al [15]	Yes	Yes	Yes	Yes	N/A		N/A	N/A
Goldberg et al [16]	Yes	Yes	Yes	Yes	N/A		N/A	N/A
Holsti et al [17]	Yes	Yes	Yes	Yes	Yes		Yes	N/A
Holt et al [18]	Yes	Yes	Yes	Yes	N/A		N/A	N/A
Ito et al [19]	Yes	Yes	Partial	Partial	N/A		N/A	N/A
Lazzara et al [20]	Yes	Yes	Yes	Yes	N/A		N/A	N/A
Marini et al [21]	Yes	Yes	Partial	Partial	N/A		N/A	N/A
Marttos et al [22]	Yes	Yes	Partial	Partial	N/A		N/A	N/A
McNelis et al [23]	Yes	Yes	Yes	Yes	N/A		N/A	N/A
Murray et al [24]	No	No	No	No	N/A		N/A	N/A
Prokazova et al [25]	Yes	Yes	Yes	Yes	No		N/A	N/A
Reynolds et al [26]	Yes	Yes	Partial	Partial	N/A		N/A	N/A
Rincon et al [27]	Yes	Yes	Partial	Partial	N/A		N/A	N/A
Rocca et al [28]	Yes	Yes	Yes	Yes	Yes		N/A	N/A
Rogove et al [29]	Yes	Yes	Partial	Partial	N/A		N/A	N/A
Ruiz-Del-Solar et al [30]	Yes	Yes	Yes	Partial	N/A		N/A	N/A
Shimizu et al [31]	Partial	Partial	Partial	Partial	N/A		N/A	N/A
Sucher et al [32]	Yes	Yes	Yes	Partial	N/A		N/A	N/A
Summerfield et al [33]	Yes	Yes	Partial	Yes	N/A		N/A	N/A
Vespa et al [34]	Yes	Yes	Yes	Yes	N/A		N/A	N/A
Wang et al [35]	Yes	Yes	Partial	Partial	N/A		N/A	N/A
Williams et al [36]	Yes	Yes	Yes	Yes	Yes		Yes	Yes
Ye et al [37]	Yes	Yes	Yes	Yes	N/A		N/A	N/A
Zeiler et al [38]	Yes	Yes	Yes	Yes	N/A		N/A	N/A

^a^N/A: not applicable.

**Table 2:** Risk of bias assessment of included studies using Standard Quality Assessment Criteria (outcomes).

Study	Outcomes						
	Outcome or exposures well defined	Appropriate sample size	Appropriate analytic methods	Variance reported	Controlled for confounding	Sufficient detail in results	Conclusions well supported
Adcock et al [6]	Yes	Yes	Yes	Yes	Partial	Yes	Yes
Alnobani et al [7]	Yes	Yes	Yes	Yes	Partial	Yes	Yes
Amodeo et al [8]	Yes	Yes	Yes	Yes	N/A^a^	Yes	Yes
Becevic et al [9]	Yes	No	Yes	Partial	Partial	Yes	No
Bettinelli et al [10]	Yes	Partial	Yes	Yes	Yes	Yes	Yes
Burke et al [11]	Partial	Partial	Partial	No	No	Partial	Yes
Duan et al [12]	Yes	Yes	Yes	No	Partial	Yes	Yes
Frazzitta et al [13]	Yes	Partial	Yes	Yes	Yes	Yes	Yes
Garingo et al [14]	Yes	Yes	Yes	Yes	Partial	Yes	Yes
Garingo et al [15]	Yes	Yes	Partial	Yes	Partial	Yes	Yes
Goldberg et al [16]	Yes	Yes	Partial	No	Partial	Yes	Yes
Holsti et al [17]	Yes	Yes	Yes	Yes	Yes	Yes	Yes
Holt et al [18]	Yes	Yes	Yes	Yes	Yes	Yes	Yes
Ito et al [19]	Yes	Partial	Partial	Yes	No	Yes	Yes
Lazzara et al [20]	Yes	Partial	Yes	Yes	Partial	Yes	Yes
Marini et al [21]	Yes	Yes	Yes	Yes	Partial	Yes	Yes
Marttos et al [22]	Partial	Partial	Partial	No	Partial	Yes	Yes
McNelis et al [23]	Yes	Yes	Yes	Yes	Partial	Yes	Yes
Murray et al [24]	Partial	Yes	No	No	No	Partial	Yes
Prokazova et al [25]	Yes	Yes	Yes	Yes	Yes	Yes	Yes
Reynolds et al [26]	Yes	Partial	No	No	Partial	Yes	Yes
Rincon et al [27]	Yes	Yes	Yes	Partial	N/A	Yes	Yes
Rocca et al [28]	Yes	Yes	Yes	Yes	Yes	Yes	Yes
Rogove et al [29]	Yes	Yes	Yes	Partial	Partial	Yes	Yes
Ruiz-Del-Solar et al [30]	Partial	Yes	Partial	No	No	Yes	Yes
Shimizu et al [31]	Partial	Partial	No	No	Partial	Partial	Yes
Sucher et al [32]	Yes	Partial	No	No	N/A	Partial	Yes
Summerfield et al [33]	Yes	Yes	Partial	Yes	Partial	Yes	Yes
Vespa et al [34]	Yes	Yes	Partial	Partial	Partial	Yes	Yes
Wang et al [35]	Yes	No	N/A	Partial	Partial	Partial	Yes
Williams et al [36]	Yes	Yes	Partial	No	Partial	Yes	Yes
Ye et al [37]	Yes	Yes	Yes	Yes	Partial	Yes	Yes
Zeiler et al [38]	N/A	N/A	N/A	N/A	N/A	Yes	Yes

^a^N/A: not applicable.

### Study Characteristics

The 33 studies included 4 categories of robotic presence from 10 different countries or regions: 18 (55%) from the United States, 3 (9%) from Canada, 2 (6%) from Italy, 2 (6%) from Japan, 3 (9%) from China, 1 (3%) from Chile, 1 (3%) from Switzerland, 1 (3%) from Saudi Arabia, 1 (3%) from Russia, and 1 (3%) from the United Kingdom. Of these 33 studies, 7 (21%) were RCTs, and 26 (79%) were observational studies. Patients were enrolled from 2007 to 2021. All studies were published in or translated to English. All studies involved patients in critical care settings, which included patients in the ICU, high-dependency unit, NICU, and emergency care settings where critical care had to be delivered. Characteristics of the included studies are shown in Table 3.

**Table 3:** Characteristics of included studies.

Study	Country or region	Study type	Setting	Population size	Use	Robot type
Adcock et al [6]	United States	Observational	ICU^a^	100 patients and 16 physicians	Telepresence	RP-7 (InTouch Health)
Alnobani et al [7]	Saudi Arabia	RCT^b^	ICU	140	Telepresence	Telemedicine Robot (Saudi Telehealth Network)
Amodeo et al [8]	Italy	Observational	NICU^c^	200 drug samples	Drug dispensing and delivery	I.V. Station (Omnicell Inc)
Becevic et al [9]	United States	Observation	ICU	5	Telepresence	RP-7 (InTouch Health)
Bettinelli et al [10]	United States	RCT	ICU	20	Telepresence	RP-7 (InTouch Health)
Burke et al [11]	United States	Observational	Emergency care	26	Telepresence	RP-7 (InTouch Health)
Duan et al [12]	China	RCT	ICU	32	Patient evaluation	MGIUS-R3 (MGI Tech Co Ltd)
Frazzitta et al [13]	Italy	RCT	ICU	40	Therapy or stroke rehabilitation	Erigo (Hocoma AG)
Garingo et al [14]	United States	Observational	ICU	46	Telepresence	RP-7 (InTouch Health)
Garingo et al [15]	United States	Observational	NICU	40	Telepresence	RP-7 (InTouch Health)
Goldberg et al [16]	United States	Observational	ICU	23 ICU bed units over a 3-year period	Telepresence	RP-7 (InTouch Health)
Holsti et al [17]	Canada	RCT	NICU	49	Therapy or stroke rehabilitation	Calmer (PCT^d^ utility patient no: CA2015/051002)
Holt et al [18]	Canada	Observational	Emergency care	38	Telepresence	RP-7 (InTouch Health)
Ito et al [19]	Japan	Observational	Emergency care	9	Patient evaluation	FASTele Tele-echography robot system
Lazzara et al [20]	United States	Observational	ICU	32	Telepresence	RP-7 (InTouch Health)
Marini et al [21]	United States	Observational	ICU	28	Telepresence	RP-6 (InTouch Health)
Marttos et al [22]	United States	Observational	Emergency care	176	Telepresence	RP-7 (InTouch Health)
McNelis et al [23]	United States	Observational	ICU	14 ICU bed units over a 2-year period	Telepresence	RP-7 (InTouch Health)
Murray et al [24]	United States	Observational	ICU	69 bed units	Telepresence	RP-7 (InTouch Health)
Prokazova et al [25]	Russia	Observational	ICU	66	Therapy or stroke rehabilitation	MOTOMed LOTTO 2 (RECK-Technik)
Reynolds et al [26]	United States	Observational	ICU	22	Telepresence	RP-7 (InTouch Health)
Rincon et al [27]	United States	Observational	ICU	34 presurvey and 40 postsurvey participants	Telepresence	RP-7 (InTouch Health)
Rocca et al [28]	Switzerland	RCT	ICU	30	Therapy or stroke rehabilitation	Erigo (Hocoma AG)
Rogove et al [29]	United States	Observational	ICU and emergency care	106	Telepresence	RP-7 (InTouch Health)
Ruiz-Del-Solar et al [30]	Chile	Observational	ICU	986 visits	Telepresence	Pudu Telepresence Robot
Shimizu et al [31]	Japan	Observational	ICU	25	Telepresence	Sota (VStone Co, Ltd)
Sucher et al [32]	United States	Observational	ICU	24 patients and 26 family members	Telepresence	RP-7 (InTouch Health)
Summerfield et al [33]	United States	Observational	ICU	23 preimplementation participants, 96 postimplementation participants, and 30 for the 2-year follow-up surveys	Drug dispensing and delivery	TUG Automated Robotic Delivery System (Aethon, Inc)
Vespa et al [34]	United States	Observational	ICU	640	Telepresence	RP-7 (InTouch Health)
Wang et al [35]	China	Observational	Isolation ward	1	Patient evaluation	MGIUS-R3 (MGI Tech Co, Ltd)
Williams et al [36]	Canada	RCT	NICU	10	Therapy or stroke rehabilitation	Calmer (PCT utility patient no: CA2015/051002)
Ye et al [37]	China	Observational	Isolation ward	23	Patient evaluation	MGIUS-R3 (MGI Tech Co Ltd)
Zeiler et al [38]	United Kingdom	Observational	ICU	10	Patient evaluation	Delica EMS 9D (Shenzhen Delica Medical Equipment Co Ltd)

^a^ICU: intensive care unit.

^b^RCT: randomized controlled trial.

^c^NICU: neonatal intensive care unit.

^d^PCT: Patent Cooperation Treaty.

### Benefits and Limitations of Robots

#### Overview

The benefits and limitations of robots can be grouped into four broad themes: (1) telepresence, (2) therapy and stroke rehabilitation, (3) patient evaluation and assessment, and (4) drug dispensing and delivery. These themes are all related to the robots’ functions in various aspects of patient care in terms of monitoring, diagnostics, and treatment.

Telepresence is defined as a technology that enables a person to perform actions at a distant location as if the person were physically present at that location. Unlike other forms of remote consultation, telepresence may also include the ability to use the medical equipment of the physician, such as stethoscopes and ultrasound, allowing physicians to remotely control the robot and interact with patients and health care personnel on site. This is different from telemedicine, which involves audio or visual communication between patients and physicians in an outpatient setting and is not the focus of this study.

Therapy and stroke rehabilitation involve interventions to treat diseases, optimize functioning or reduce disability in individuals. Patient evaluation involves assessing a patient’s current condition to identify health problems and plan treatment. Finally, drug dispensing and delivery involve the process of preparing and providing medicine to a patient based on a health care provider’s prescription.

#### Theme 1: Telepresence

Approximately 64% (21/33) of studies identified 5 different telepresence robots. RP-7 (InTouch Health) [6,9-11,14-16,18,20-24,26,27,29,32,34] was the main robotic telepresence system used in 55% (18/33) of studies (RP-6 was used in one). RP-7 has a bidirectional audio and video communication system that displays real time video and camera systems. Devices such as electronic stethoscopes, otoscopes, pulse oximeters, and ultrasound probes can be connected to the expansion bay of a robot to transmit medical data. The robotic system can be remotely controlled and monitored by physicians. RP-7 can also be linked to and automatically acquire information from hospital-based electronic data systems.

Sota (Vstone Co Ltd) [31] is a bedside AI-enhanced robot capable of alerting physicians about anomalies in biological information. Such information can be derived in real time from bedside monitors or existing electronic health records. The Sota robot alerts physicians through voice warning systems coupled with alarms. In addition to the alert function, it can function as a social robot by responding to simple voice commands. In contrast to Sota, Pudu [7] is a social robot designed specifically to provide telepresence and communication services and deliver emotional and mental care to isolated patients with COVID-19. It works by using an assistive teleoperation mode, allowing for remote control of the robot’s movements using an Xbox (Microsoft) controller joystick. The robot comprises smooth surfaces and fulfills health requirements, where it can be sanitized in a safe and efficient way. Two unnamed telepresence robots were used in a study in Saudi Arabia [30]. These 2 robots had similar functions and equipment to RP-7.

Patients benefited from telepresence because of the reduced response time [11,14,18,21,24,29,34] by as much as 95.8%, allowing earlier intervention, higher patient survivability, and lower mortality rates [16,21]. Unlike more traditional methods, the physician was able to have a realistic physical presence and interact directly with ICU staff and patients at the bedside [14]. Mortality and complication rates could be reduced by 25% [21] to 59% [16], especially at night when there were often staff shortages [16]. This was especially pertinent in time-sensitive settings such as trauma with a short time window to intervention [34] and rural hospitals with poor access to specialist physicians [22,24,26]. Overall, telepresence allowed care to be provided in a timely manner regardless of the location of the physician or the time of day.

When compared with care delivered by traditional methods, the studies that measured rates of ICU admission and the average length of stay consistently showed a decrease in length of stay compared with both conventional rounding and telephone rounding, ranging from 6.25% [24] to 33% [16], and an increase in appropriate ICU admissions rates [16,21,23,24,34]. With prompt response time and closer monitoring, the rates of developing significant complications were lower. Any emergencies or acute changes were tended to before significant health repercussions developed [6].

The usual standards of care and assessment were not compromised when the robots were used. Approximately 12% (4/33) of studies mentioned that the RP-7 robot was able to perform a good range of tasks, including physical examinations, with a similar level of accuracy and precision compared with traditional methods of care, allowing the physician to come to an accurate clinical conclusion [6,11,14,21].

For hospitals, there was a financial benefit from direct cost savings as robotic presence reduced the need to employ full-time staff for ward rounds during off-peak hours [16,23,34]. There were also cost savings from faster patient turnover and the lowered external transfer rate of rural hospitals [28]. By reducing the number of external transfers, the number of unnecessary admissions to hospitals was reduced. In total, the financial benefits were as much as US $1.1 million per year [34].

Robots were well-received by patients, family members, and staff [7,9,10,15,20-23,26,27,31]. Despite a telepresence robot providing remote physician presence, patients did not perceive the physician to be caring less or compromising the quality of care [7,15,29,32]. Staff had an overall positive perception of telepresence robots, including in areas such as usability [7], acceptability [22], efficiency, communication [9], and decreased noise or traffic in ICUs during the morning rounds [32]. For example, in a study by Alnobani et al [7], 71.5% of staff felt that the robot saved time, and 77.2% of staff felt that it improved clinical diagnosis.

Robots also played a role in the education and mentoring of staff [21,26]. Staff education included mentoring nurses, discussing admission and discharge issues, and facilitating compliance with treatment protocols. Expert opinions from nurses and physicians were more accessible for direct guidance of resuscitation efforts, even in remote areas [26]. Interactivity and 2-way communication were preserved during the teaching that occurred during remote rounding [21]. In addition, hospital psychologists used the Pudu robot [30] to provide remote emotional and mental care in the COVID-19 ward. All patients who received such psychological care via Pudu showed positive attitudes and emotions. Patients and family members were satisfied with how Pudu enabled their interactions to be extended and uninterrupted, providing them with good emotional support.

Limitations of telepresence included discrepancies between on-site and off-site evaluations, although these could be attributed to subjective differences [14]. One of the studies reported limitations in determining abdominal distension and capillary refill time and using an electronic stethoscope for heart, breath, and bowel sounds [14]. However, the study also mentioned that these discrepancies were present between 2 bedside neonatologists, thus rendering it possible that these differences in findings were inherently subjective. Another study reported limitations in accurate assessments using the Mayo Full Outline of Unresponsiveness scale, particularly for brainstem and pupillary responses [6]. However, the study also reported that the Glasgow Coma Scale was a good alternative that was accurately assessed using the telepresence robot.

Medicolegal challenges existed, such as a lack of established protocols causing regulatory barriers in terms of obtaining credentialing and malpractice liability [7,29], as well as financial barriers in terms of patient billing and difficulty obtaining reimbursement [29]. In addition, hidden costs for maintenance and electricity, licensing, technical issues, and space constraints acted as barriers to use [29].

Although many studies mentioned a reduction in face-to-face response time, 6% (2/33) of studies reported an *increase* in time spent on patient encounters, attributed to the time taken to operate and maneuver the robot, as well as to resolve technical issues such as internet connectivity problems [15,23]. Similar technological limitations of internet connectivity and maneuvering difficulty were also reported in another study [14]. Fortunately, most incidents of poor connectivity were promptly overcome within 5 minutes. Additional technological difficulties included poor audio quality because of transmission of ambient noise and poor angle of visibility when attempting to view the thoracoabdominal area [22].

In terms of staff perception, some concerns were raised with regard to the impact of robot use. These were in the areas of threat to staff job security and additional responsibilities [7]. The staff also raised some issues with regard to patient confidentiality, patient privacy, and legal liability. Nonetheless, although these concerns existed, there was general acceptance and approval of telepresence technology among the staff surveyed [7]. The benefits and limitations in the field of telepresence are summarized in Textbox 1.

**Theme 1: robotic telepresence.**

**Robot examples**

- RP-7 (InTouch Health): 18 papers [6,9-11,14-16,18,20-24,26,27,29,32,34]
- SotaTM (VStone Co Ltd): 1 paper [32]
- 2 unnamed telerobots: 1 paper [31]
- Pudu Telepresence Robot: 1 paper [30]

**Benefits**

- Patient survival and patient mortality rate [16,21]
  - 59% lower mortality rate [16]
  - 25% decrease in mortality from robotic telerounding vs conventional rounding [21]: 12% (5/42) vs 16% (6/37); *P*=.75
- Provides superior care to alternatives [18,23]
  - Higher average number of therapeutic interventions vs telephone rounding [23]: 5.3 (SD 1.7) vs 1.3 (SD 1.4); *P*<.01
  - Less overnight calls and less unexpected events vs telephone rounding [23]: 0.1 (SD 0.2) vs 1.3 (SD 0.5); *P*<.05
  - Reduced external transfer rate by 63%, allowing patients to be effectively treated in local clinics [18] and receive specialist care closer to home and earlier stabilization
  - Patient care time can be lengthened to allow for extended interaction with family members for those under isolated care without risk of contagion exposure [30]
- Reduction in face-to-face response time, leading to earlier intervention and access to specialists [11,14,18,21,24,29,34]
  - Response latency in robotic telepresence vs conventional care [34]
    - To routine and urgent pages: 9.2 (SD 9.3) minutes vs 218 (SD 186) minutes; *P*<.001
    - To brain ischemia: 7.9 (SD 2.8) minutes vs 152 (SD 85) minutes; *P*<.001
    - To elevated intracranial pressure: 11 (SD 14) minutes vs 108 (SD 55) minutes; *P*<.001
- Decreased intensive care unit length of stay [16,21,23,24,34]
  - Length of stay in intensive care unit decreased; response latency in robotic telepresence vs conventional rounding:
    - 7.5 (SD 8.8) days vs 8 (SD 8.3) days [34]
    - 33% reduction [16]
    - 2.5 days vs 3.3 days [24]
    - 5 (SD 2) days vs 6 (SD 3) days; *P*=.57 [21]
  - Length of stay in intensive care unit decreased; response latency in robotic telepresence vs telephone rounds [23]: 4.8 (SD 2.6) days vs 5.6 (SD 2.2) days; *P*<.05
  - Length of stay in hospital decreased; response latency in robotic telepresence vs telephone rounds [23]: 10.2 (SD 4.3) days vs 12.3 (SD 4.4) days; *P*<.05
- Financial benefit: decreased cost, increased revenue, lower start-up costs or flexibility, and no need to employ full-time staff such as in the central monitoring model [16,18,34]
  - 29% lower adjusted mean direct cost estimated per case [16]
  - US $1.1 million cost savings over 1 year [34]
  - CAD $360,000 (US $285,420) savings over the study period [18]
    - Cost of round trip, cost of hospital stay, and miscellaneous costs such as family transport and accommodation
- Does not compromise on usual standard of care and assessment consistency between bedside and remote examination [6,11,14,21,30]
  - Bedside vs remote examination [6]
    - Mean Glasgow Coma Scale: 7.5 (SD 3.67) vs 7.23 (SD 3.85), difference 0.25 (SD 0.10); *P*=.01; however, the difference is not clinically significant; Pearson correlation coefficient=0.97
    - Mean Full Outline of Unresponsiveness: 9.63 (SD 4.76) vs 9.21 (SD 4.74), difference 0.40 (SD 2.00); *P*=.05; Pearson correlation coefficient=0.91
  - Agreements in most physical examination assessments between both on-site and off-site neonatologists [14]
- Education benefits [21,26,34]
  - Educational experience of medical students, physician assistants, and surgical residents not affected by response latency in robotic telepresence [21]; average Likert score:
    - Surgical residents: 4.5 (SD 0.2); *P*>.05
    - Medical students: 3.9 (SD 0.4); *P*>.05
    - Physician assistants: 4.4 (SD 0.4); *P*>.05
  - 87% felt that it improved nursing education [26]
- Positive staff perception: usability, acceptability, efficiency, communication, and decreased noise or traffic [7,9,10,15,20-23,26,27,30,31]
  - Positive health care worker attitude toward telepresence [7]
    - Increasing communication and collaboration among providers: 4.01/5 (SD 0.800)
    - Improve clinical decisions: 3.91/5 (SD 0.877)
    - Provide access to specialized second opinion consultation: 4.19/5 (SD 0.774)
    - Facilitates diagnosis and treatment: 3.87 (SD 0.847)
  - Collaboration and Satisfaction About Care Decisions survey increased [10]
    - RP-7 vs baseline: 51.3 vs 43.0; *P*=.01
    - Robot rounds vs telephone rounds: 51.3 vs 50.5; *P*=.30
  - Higher user satisfaction vs telephone rounds [23]: 7.7 (SD 2.3) vs 5.6 (SD 2.1); *P*<.01
  - Night nurses’ perceptions [27]:
    - Intensive care unit physicians sufficiently available: 6%-20%; difference in proportions 14%; *P*=.008
    - Present during acute emergencies: 44%-65%; difference in proportions 21%; *P*=.007
  - SotaTM: alerts issued by the robot to warn of detected anomalies perceived to be more effective than the current desktop-based system [31]
- Patient, family perception, or satisfaction [15,26,32]
  - 100% viewed it as valuable in improving family and patient satisfaction [26]
  - 100% of parents felt comfortable talking to off-site neonatologists on a mobile robot [15]
  - 84% believed that care was better as the robot was used [32]
- Alleviate future staffing shortages
  - Allow for redistribution, easing the overcapacity issues that strain tertiary care centers [18]

**Limitations**

- Lack of established protocols [7,29]
  - Hindered by regulatory barriers of licensing, credentialing, and malpractice protection [29]
  - Increases legal liability challenges [7]: 2.66/5 (SD 0.784)
- Hidden costs [29]
  - Finance barriers of miscellaneous costs, billing, and reimbursement issues [29]
- Discrepancies between on-site and off-site evaluations for physical findings [7,14]
  - Poor agreements on physical examination parameters (breath, heart and bowel sounds, and capillary refill time) [14], although they also occurred regardless of response latency in robotic telepresence use between 2 on-site physicians
- Decreased efficiency and longer time spent on patient encounters
  - Time spent [15] off-site vs on-site neonatologist: 8 (IQR 7-10.5) minutes vs 5 (IQR 5-6) minutes; *P*=.002; difference because of time needed to operate and maneuver robot or slower or dropped internet connection
  - Longer rounding time [23] response latency in robotic telepresence vs telephone: 33.2 (SD 15.4) minutes vs 18.3 (SD 12.7) minutes; *P*<.05
- User-dependent experience required training [7,21]
- Technological limitations
  - Difficulties maintaining internet connection in 23% encounters; 93% reconnected in <5 minutes [14]
  - Average of 2.1 (SD 1.2) interruptions per session because of wireless signal loss [23]
- Ethical challenges [7]
  - Threatens patient’s confidentiality: 2.96/5 (SD 0.955)
  - Raises privacy concerns: 3.12/5 (SD 0.956)
- Poor staff perception [7,9,20,21,26,31]
  - 50% of physicians did not think physician quality of life improved [26]
  - Did not meet nurses’ expectations [21]; Likert score of 3.5 (SD 1.0)
  - Threatens staff position [7]: 3.09/5 (SD 0.925)
  - Increases staff workload [7]: 3.09/5 (SD 0.925)
  - Creates new responsibilities for staff [7]: 2.74/5 (SD 0.940)
  - Only 20% of nursing respondents were satisfied with the quality of technology of Sota Robot [31]

#### Theme 2: Therapy and Stroke Rehabilitation

Approximately 15% (5/33) of studies identified 3 different robots (Table 4) that provided various forms of therapy or rehabilitation in the intensive care context. They played a role in enhancing and optimizing the process of patient recovery. Of the 3 robots we identified, 2 (67%) were targeted toward early functional rehabilitation for patients with stroke [13,25,28], and 1 (33%) was a robot specially designed for the care of neonates in the NICU [17,36]. Physiological parameters were measured to evaluate the effects of the 3 robots, which have been shown to be beneficial overall.

MOTOmed LOTTO 2 (RECK-Technik) [25] is a robotic movement therapy device that enables leg mobilization in a supine position, allowing for passive, active, or assisted mobilization for patients on prolonged bed rest. Early rehabilitation of patients of stroke has been shown to lead to better functional outcomes in patients with acute ischemic stroke [39]. MOTOmed [25] achieved better outcomes than a standard care protocol in terms of recovery of neurological function. MOTOmed also achieved a lower incidence of severe multicomponent multiple organ dysfunction (14% vs 41%; *P*<.05; intervention vs control) and pulmonary embolism (12% vs 33%; *P*<.05; intervention vs control). In patients with neurological pathologies, MOTOMed stimulated the sympathetic system, which helped recovery by preventing polyneuropathy and improving awareness of disorders of consciousness. However, MOTOMed should be used with caution in patients with subarachnoid hemorrhage, as catecholamine overproduction as a stress response was associated with complications such as the increased risk of vasospasm [28].

Erigo (Hocoma AG) [13,28] is a robot that combines a tilt table with a leg movement system, allowing for progressive and customizable verticalization of patients with acquired brain injury. The gradual mobilization in Erigo overcame an important limitation to early mobilization, which was orthostatic intolerance [13,28]. Orthostatic hypotension with compensatory sympathetic catecholamine production was reduced most significantly with Erigo compared with other forms of early mobilization, namely conventional in-bed physiotherapy and MOTOMed [28]. Therefore, it could be safely used in patients with subarachnoid hemorrhage. Compared with in-bed physiotherapy, Erigo produced statistically significant, higher improvements in the Coma Recovery Scale (17.0 vs 5.0; *P*=.03; intervention vs control) and Disability Rating Scale (−20.0 vs −6.0; *P*=.04; intervention vs control) [13]. It also produced nonstatistically significant improvements in the Glasgow Coma Scale and levels of cognitive functioning. However, a longer ICU stay was required to complete the verticalization protocol before transfer to a neurological rehabilitation unit [13].

Calmer [17,36] is a robot used in the NICU, which is designed to reduce pain in preterm infants subjected to multiple painful procedures. Calmer simulates skin-to-skin holding via touch, breathing motions, and sound stimulation. Calmer’s artificial skin-like surface and vertical movement mimic breathing motion and heartbeat sound to match those of infants’ mothers. Compared with the standard care of facilitated tucking, Calmer reduced preterm infant pain reactivity. Approximately 6% (2/33) of studies consistently showed that infants had greater *para*sympathetic activation and hence greater physiological stress *reduction* during painful procedures such as blood taking [36]. Calmer was a safe, ergonomic, and cheaper alternative to the manpower-intensive facilitated tucking. Research is ongoing to incorporate Calmer into incubators, which would potentially allow for cost savings of as much as US $380,000 per year in a 60-bed NICU [17]. The benefits and limitations of the 3 abovementioned robots are summarized in Table 4.

**Table 4:** Theme 2: therapy or stroke rehabilitation.

Robot examples	Benefits	Limitations
Calmer: 2 papers [17,36]	Efficacy in reducing infant pain Increases HF^a,b^ component (parasympathetic activity) of HRV^c^ (Hz/ms2) [36], Calmer vs standard FT^d^ group: Baseline (before procedure): 36.0 (23.7-73.2) vs 3.6 (3.1-9.1) Poke (during painful procedure): 2.2 (1.1-3.0) vs 0.4 (0.3-7.2) Recovery (post procedure): 6.8 (1.7-21.1) vs 5.2 (4.1-12.8) Difference BIIP^e^ score in peak pain phases, Calmer vs FT [17]: 4.0 (SD 2.7) vs 3.2 (SD 2.7; 95% CI −0.45 to 2.72) Cost savings US $380,000 per year in 60-bed NICU^f^ [17] No safety issues with short-term use	Nil mentioned
MOTOmed LOTTO 2 (RECK-Technik): 1 paper [25]	Safe for early rehabilitation of patients of stroke who are critically ill Better outcomes in stroke rehabilitation (day 21 after stroke), intervention vs control group: Neurological outcomes improved GCS^g^: 15 (14-15) vs 15 (15-15); *P*=.32 NIHSS^h^: 11 (8-25) vs 15 (12-19); *P*>.05 APACHE^i^ 2: 6 (3-14) vs 9 (6-12); *P*>.05 Complications Incidence of MOD^j^: 60% vs 67%; *P*>.05 Incidence of severe MOD: 14% vs 41%; *P*<.05 MOD scale 0 (0-1) vs 1 (0-2); *P*>.05 Incidence of PE^k^: 12% vs 33%; *P*<.05 Incidence of death from PE: 0 vs 1/3 Mortality rate decreased, intervention vs control group: 12% vs 39%; *P*<.05	No significant changes in DVT^l^ incidence, intervention vs control group: DVT incidence 58% vs 45%; *P*>.05
Erigo (Hocoma AG): 2 papers [13,28]	Better clinical outcomes—greater difference in neurological scoring systems Difference in values at ICU^m^ admission and at rehabilitation discharge [13], intervention vs control: No orthostatic intolerance occurred DRS^n^: −20.0 (−22.0 to −4.5) vs −6.0 (−12.7 to −2.0); *P*=.04 CRSr^o^: 17.0 (5.1-18.8) vs 5.0 (2.4-11.0); *P*=.03 GCS: 7.0 (3.2-10.0) vs 4.5 (3.0-6.5); *P*=.08 LCF^p^: 4.0 (1.0-5.0) vs 2.5 (1.0-4.0); *P*=.14 No increase in catecholamine production [28]	Longer LoS^q^ in ICU [13], intervention vs control group: 38.8 (SD 15.7) days vs 25.1 (SD 11.2) days; *P*=.01 To complete stepping verticalization protocol before being moved to the neurological rehabilitation unit

^a^HF: high frequency.

^b^Indicates parasympathetic activity: decreased HF=stress; increased HF=calmness or stress recovery.

^c^HRV: heart rate variability.

^d^FT: facilitated tucking.

^e^BIIP: Behavioural Indicators of Infant Pain.

^f^NICU: neonatal intensive care unit.

^g^GCS: Glasgow Coma Scale.

^h^NIHSS: National Institutes of Health Stroke Scale.

^i^APACHE: Acute Physiology and Clinical Health Evaluation.

^j^MOD: multiorgan dysfunction.

^k^PE: pulmonary embolism.

^l^DVT: deep vein thrombosis.

^m^ICU: intensive care unit.

^n^DRS: Disability Rating Scale.

^o^CRSr: Coma Recovery Scale.

^p^LCF: levels of cognitive functioning.

^q^LoS: length of stay.

#### Theme 3: Patient Evaluation and Assessment

Approximately 15% (5/33) of studies identified 3 different robots (Table 5). These robots were used to evaluate various parameters of patients, including patient monitoring in a critical care setting and ensuring quality evaluation from a remote location. Robots also used ultrasound systems to enhance their evaluation capability [12,19,35,37,38].

FASTele [19] is a wearable, portable, attachable tele-echography robot system for focused assessment with sonography for trauma (FAST) scans. FASTele [19] was able to produce sharp ultrasound images of all FAST areas, even under maximum vehicle acceleration, in all axial directions, and under various body motion conditions, applicable to a range of body types. However, a longer time was required to perform FAST, especially in patients who were overweight, as it required attaching a corset to each FAST area. Patients were also at risk of injury during the attachment of the robot system.

MGIUS-R3 (MGI Tech Co Ltd) [12,35,37] is a 5G-powered, remote, robot-assisted teleultrasound diagnostic system. It combines a robotic arm, an ultrasound imaging system, and audio-visual communication for teleoperation. Application of MGIUS-R3 in cardiopulmonary assessment achieved image quality acquisition, labeling, and analysis equivalent to that of traditional ultrasound, enabling accurate diagnosis [12,37]. No complications or delays were noted during the image acquisition process. It performed satisfactorily even at remote distances of 700 km [35]. Overall, there was a higher level of safety because of the reduction in infection risk for patients and physicians. The patient would not be exposed to cross-infection during transport to the radiographer’s room in the hospital, and the physician would not be exposed to a patient with an infectious disease [12]. However, the robotic arm faced difficulties in reaching some body parts [12,37]. In addition, the ultrasound frequency was limited as the robot had only 1 convex array probe, thus affecting the quality of cardiac images [12,37].

Delica EMS 9D (Shenzhen Delica Medical Equipment Co Ltd) [38] is a portable transcranial Doppler (TCD) system for simultaneous bilateral middle cerebral artery blood flow velocity recording. It comprises Doppler ultrasound probes attached to a robotic drive supported by a headband frame. The robot performs the automated functions of scan, search, direction, and track. Compared with standard TCD systems, Delica achieved improved image-capturing capabilities without interruption or the need for manual adjustments [38]. Delica also reduced the risk of disrupting other in situ monitoring, making it highly applicable to the critical care setting. Overall, this led to time saving and increased efficiency. However, this device has some technological limitations. The only available signal recording frequency was 100 Hz, and it could not perform heart rate variability analyses requiring frequencies of ≥200 Hz. A possible safety concern involved increased intracranial pressure in one patient, which was resolved with headband readjustment. The benefits and limitations of the 3 abovementioned robots are summarized in Table 5.

**Table 5:** Theme 3: patient evaluation.

Robot examples	Benefits	Limitations
FASTele: 1 paper [19]	Extracted echo images met and exceeded the defined FAST^a^ criteria Brightness gradient of echo images vs values required by the physician: 4.7 (SD 10.4) vs 3.9 (SD 9.8) FAST performance achieved with vehicle motions: at maximal acceleration in all axial directions and body motion conditions Constant pressure to hold the probe is not required	Likelihood of longer time to perform FAST: requires attaching a corset to each FAST area and may cause possible injury to patients Prolonged wrapping time in patients who are overweight System to be improved for medical physicians to operate it easily
MGIUS-R3 (MGI Tech Co Ltd): 3 papers [12,35,37]	Clear images: image quality score 4.73 (high quality) [12] Comparable diagnostic results to bedside examination [12,37] Safety [12,37] Able to complete an assessment successfully as per established examination protocol [12,37] No need to transport patients who are clinically ill for assessment and minimizes radiographer and hospital exposure to COVID-19 and other infectious diseases [12,37] Able to be used in isolation wards [37] Multiple protection measures [37] Simultaneous start prompts Emergency stop button Speed limit settings on the robotic arm Faster [12,37] No delay in scanning, 10-20 minutes per examination [37] 5G network system: ensures real time US^b^ image; detailed physician-patient communication, 20 times better transmission rate; delay reduced by a factor of 10, allowing high-definition and accurate video transmission [12,37] Able to perform from a remote distance of 700 km away [35]	Difficulty of the robotic arm in reaching some body parts, especially in patients who are critically ill [37] and on the patient’s side [12] Required mobilization of intubated COVID-19 patient for AP^c^ and lateral thoracic views [35] Only one convex array probe—frequency limitation and unable to scan heart [12,37] 15.6% inconsistent results between robot-assisted teleultrasound and bedside ultrasound [12] Difficulty in 3D space perception, requiring practice and familiarization [12]
Delica EMS 9D robotic TCD^d^ (Shenzhen Delica Medical Equipment Co Ltd): 1 paper [38]	Improved image-capturing capability vs standard TCD systems Continuous, uninterrupted recording for 4 hours Better image quality Flow velocity signals are accurately captured even in the presence of other in situ multimodal monitoring devices Allows multimonitoring in moderate to severe TBI^e^ patients Reduces risk of disruption of monitoring from repeated loosening and manipulation of other devices Increased efficiency from time saved in manual adjustment of the probe, which is crucial in patients who are critically ill Increased patient comfort and fast turnover with easy cleaning of the device	Scan and track functions are less functional Limitation in available signal recording frequency (100 Hz only) Potential complications of raised ICP^f^

^a^FAST: focused assessment with sonography for trauma.

^b^US: ultrasound.

^c^AP: anterior posterior.

^d^TCD: transcranial Doppler.

^e^TBI: traumatic brain injury.

^f^ICP: intracranial pressure.

#### Theme 4: Drug Dispensing and Delivery

Approximately 6% (2/33) of studies identified 2 different robots (Table 6). Both robots were involved in drug dispensing or delivery, and both showed time reduction, cost savings, and increased precision in drug preparation.

The TUG Automated Robotic Delivery System (Aethon Inc) [33] is a robot affixed to a medication delivery cart controlled by pharmacy staff. When a medication delivery was planned, a pharmacy staff member summoned the robot, inputted the desired sequence of deliveries, and loaded the medications onto the robot. The robot then traveled to the desired locations where the nurses unloaded it. The robots delivered most medications except for *stat* medications meant for immediate administration and controlled drugs. The TUG robot reduced the mean pharmacy cycle time from order receipt to order exit by 29.6% [33]. The technician delivery time decreased by 7.2 hours, and the saved time was used in handling other pharmacy tasks, leading to significant cost savings of an estimated US $14,100 yearly. It was well-received by nurses and pharmacists in terms of reliability and performance. However, nurses were dissatisfied that they now had to sort and store medications, which had been previously performed by technicians. There was also a downtime of robots because of infrastructure and robot-related problems such as power supply and cart issues.

I.V. Station (Omnicell Inc) [8] is a fully automated robot that prepares sterile injectable drugs. It performs all stages of preparation, from reconstitution to dilution and final preparation. I.V. Station achieved increased precision in drug preparation compared with manual preparation [8]. Patient adverse effects from overdosing and loss of drug efficacy from underdosing were reduced. In addition, there were fewer potentially harmful staff events. Precision is especially crucial for preterm neonates who require complex therapy and are at high risk of fatal medication errors [8]. A decrease in the cost and mean preparation time by as much as 8% and 2 hours 57 minutes, respectively, was achieved during the preparation of greater quantities. Time savings enabled a focus on other aspects of care, including engaging and educating families. However, when preparing smaller quantities, the robot was more expensive and slower than manual preparation. In addition, mechanical or software failure events affected the workflow and caused medication wastage. The benefits and limitations of the 2 abovementioned robots are summarized in Table 6.

**Table 6:** Theme 4: drug dispensing and delivery.

Robot examples	Benefits	Limitations
TUG Automated Robotic Delivery System (Aethon Inc): 1 paper [33]	Increased efficiency of medication delivery before implementation vs 2 years after implementation Mean total mean pharmacy cycle time (order receipt to order exit): 73.9 (SD 2.21) minutes vs 52 (SD 28.6) minutes Mean time for label printing, 13.1 (SD 3.9) minutes vs 7.4 (SD 4.1) minutes Mean idle time for medication delivery: 27.3 (SD 8.2) minutes vs 15.3 (SD 8.4) minutes Time and cost savings 7.2 hours of technician time saved Projected annual cost savings: US $14,100 Positive nurse perceptions: Perception before implementation vs post implementation: General satisfaction increased; *P*<.02 Robot reliability increased; *P*<.01	Limited benefit in timeliness and perceived quality of delivery service Decreased efficiency in nondelivery aspects—nurses have additional duty to sort and store delivered medications. Low robot reliability perceived by technicians that improved at 2-year follow-up
I.V. Station (Omnicell Inc): 1 paper [8]	Better clinical outcomes Increased precision in drug preparation vs manual preparation: accuracy within 5% to –5% Improved safety for both patient and staff Increased efficiency during the preparation of higher dose quantities Range: time savings of 16 seconds (acyclovir) to 2 hours 57 minutes (teicoplanin) Reduced costs during the preparation of higher dose quantities Range: 8% (ampicillin) to 66% (teicoplanin)	Mechanical or software failure events Decreased efficiency during the preparation of lower dose quantities Increased costs during the preparation of lower dose quantities Hidden costs (not included in cost calculations) Electricity Machine maintenance Days of downtime because of machine failure However, the inactivity rate was low at 2.5% (9.5/365 days)

## Discussion

### Benefits of Robots

Our review demonstrates the numerous beneficial capabilities of robots. We found that the greatest application of robots in critical care was in telepresence, and the most studied telepresence robot was RP-7. Overall, the evidence showed that robots were beneficial and well-received and delivered significant patient, staff, and hospital benefits. The abovementioned robots covered various aspects of ICU care. Some were used during acute settings, such as telepresence robots for urgent consultations or for patient evaluation. Meanwhile, the robots that focused on rehabilitation or drug dispensing were more directed toward general functioning and processes in the ICU.

In terms of efficiency, robots in the areas of telepresence, patient evaluation, and drug dispensing and delivery were able to provide time savings. In the critical care setting, this was especially important, as face-to-face response time could be reduced, allowing patients to have faster access to specialists.

Similarly, there were cost savings in the applications of telepresence, therapy or rehabilitation, and drug dispensing or delivery. Although the amount saved varied across different studies, with the highest being US $1.16 million reported by Vespa et al [34], all studies agreed that cost savings were beneficial to hospitals.

Robots could outperform current care standards and supplement human efforts in the fields of telepresence, therapy, and patient evaluation. For example, Delica TCD [38] allowed for improved Doppler image capturing that a normal TCD could not achieve with manual effort. With Erigo [13,28], concurrent verticalization with stepping eliminated orthostatic hypotension, which previously prevented early mobilization post acquired brain injury. This enabled improved care for patients with a subsequent reduction in mortality rate.

The workload of physicians could also be alleviated using robots. RP-7 [6,9-11,14-16,18,20-24,26,27,29,32,34] supplemented rounding and was used during off-peak hours, reducing the need for physical physician presence during graveyard shifts. This is particularly relevant during the current COVID-19 pandemic, where physicians must grapple with a heavy workload [40]. Physicians could then focus on more holistic patient care, including psychological and social aspects.

A benefit mentioned across all themes was safety. Generally, papers in each theme agreed that robots were able to either meet current safety standards by providing diagnoses comparable with those of existing standardized methods or further reduce risks, for example, by improving the precision of medication preparation [8]. In more recent papers published in 2020 and 2021, a consistent theme was that robots could allow medical professionals to maintain social distancing while still effectively treating patients. This prevented exposure to pathogens and also reduced the use of disposable personal protective equipment.

Many believe that robots are unequipped to handle soft skills instrumental in health care. Although robots cannot counsel a patient or console a distressed family member, they can nonetheless emulate the human touch in their own unique ways. For example, Calmer [17,36] sought to mimic human touch without the intention to *replace the parent*. The technology is a step in the right direction, and the comfort that the robot brings to infants could potentially be extended to the care of adult patients who are vulnerable and critically ill as well.

### Limitations of Robots

Although robots could help reduce the workload in some areas, they could lead to both human unemployment and overreliance on robots. Although robots cannot fully replace physicians, they can and already have replaced some manpower in the health care sector. When surveyed, staff in the ICU felt that their jobs were moderately threatened [7].

Another concern was the possibility of hacking. Some robots such as RP-7 and MGIUS-R3 relied on Wi-Fi or 5G and thus were susceptible to security issues and data breaches. If robots were to break down or encounter technological issues, systems must be in place to immediately recognize and mitigate these issues, given that time is always of the essence in health care. Otherwise, cybersecurity breakdowns would lead to workflow disruptions, loss of patient privacy, and significant medicolegal repercussions [7].

Robot use may translate to increased costs for patients because of the cost of robots, licensing, installation, maintenance, and repairs. In addition, because of the current lack of legislation regarding billing for services rendered by robots, hospitals may excessively charge for robotic use. Goldberg et al [16] reported that although the mean cost estimates per case decreased by 29%, the billing charges instead increased by 70% [16]. Overall, this could mean that although costs for hospitals decreased, costs for patients ultimately increased, which also reflects a mismatch in expected outcomes, possibly because of a lack of existing price controls.

We must also recognize that telepresence implementation may be more suited to hospitals that already have an effective ICU staffing infrastructure. Although the ideal aim of telepresence is to relieve the workload of ICUs with scarce resources, it may potentially create a paradoxical imbalance in resource allocation, where staff from underresourced ICUs are drawn to larger, more established ICUs that can sustain telepresence.

Although robots complement and aid in workload, leading to generally positive perceptions, some robots were less enthusiastically received. This could be attributed to differences in the ease of use of the robot, the context of their application, and baseline perceptions. One of the studies mentioned that nurses still believed that the physical presence of intensivists was preferable and necessary. In any case, the role of telepresence is not to completely replace physical physician presence but to supplement staffing during off-peak hours, ensuring safe coverage.

As robotic intervention becomes more prevalent and integrated into health care, this necessitates a conversation around developing an ethical and legal framework with regard to accountability. With exponential digital growth over the past century, we will certainly continue to see an increasing overlap between the physical and digital worlds. It is imperative to form strict lines of accountability—shall it lie with the physician who used it, or is the robot’s developer and manufacturer who should be held accountable for any errors?

### Further Applications

Among the excluded papers, promising potential applications of robots were shown, as elaborated in the following sections.

#### The COVID-19 Pandemic

In the past 2 years, research has been greatly focused on the COVID-19 pandemic. As many papers have yet to have formal trials on patients, they were excluded based on our criteria. However, they demonstrate highly applicable uses in the critical care setting. Overall, 7 COVID-19-related papers echoed similar themes to those papers included in our systematic review. In addition, by enabling remote disinfection or control of equipment, robots reduced the exposure of medical staff to pathogens and lowered the use of personal protective equipment.

Approximately 29% (2/7) of papers described the use of UV-C disinfectant robots within the ICU [41,42]. Choi et al [41] described a UV light-emitting diode robot (UVER-SR1, UVER Co) [41] with a freely rotating arm. It could successfully disinfect ICU rooms. Another mobile UV-C robot (ASSUM, Assum Tech) [42] similarly demonstrated a 99.91% reduction in the SARS-CoV-2 load within a few minutes. Overall, the 2 robots worked to reduce the exposure of cleaning and health care personnel to contaminated surfaces.

Approximately 57% (4/7) of papers described the use of robotics to reduce the need for health care staff to physically enter patient rooms. Sawyer (Rethink Robotics GmbH) [43], a 7-axis robot with flexible joints and a camera, could successfully perform a variety of COVID-19 health care tasks: intravenous pump device continuation, ventilator knob adjustment, ICU monitor silencing, oxygen knob adjustment, and call button deactivation. Vagvolgyi et al [44] demonstrated the use of a telerobotic cartesian system to allow the adjustment of ventilator settings from outside the ICU. Similarly, this was feasible in a simulated ICU environment and specifically saved 59.8% of the time (a decrease from 271 seconds to 109seconds). A 4-Degrees-Of-Freedom [45] robot was also able to interact with the touchscreen instrument panel of dialysis machines, achieving fast and simple control of the machine in the context of emergency dialysis. Finally, in a newly published study, a team from the Massachusetts Institute of Technology developed Emergency-Vent [46], a robotic gripper that automated the task of manually squeezing a resuscitator bag. The robot was able to customize ventilator settings within each cycle of breathing, including tidal volume, respiratory rate, inspiration-expiration time ratio, positive end–expiratory pressure, and assist control trigger threshold. It successfully ventilated a porcine model and performed comparably to that of an experienced anesthesiologist manually pumping the resuscitator bag. Both ease and cost of use were low. Such robotic ventilator technology is especially relevant in the context of the COVID-19 pandemic, as manpower and ventilator shortages lead to the need for cheap ventilator alternatives.

Long-term bed immobilization limits the recovery of patients with COVID-19 and puts them at risk of many complications such as pressure sores, contractures, and joint immobility. It is especially challenging to manage the positioning of patients with COVID-19, given that multiple devices and equipment surround the bed and that medical staff are at greater risk of infection with increased frequency of patient contact. A robotized hospital bed [47] was designed with a flexible mattress and an easily sanitized structure that allows the mobilization of major joints. This approach has several benefits. First, the effective mobilization of patients prevented the accumulation of secretions in the lungs. A self-movable bed that inclines can counter mucus engorgement and subsequent atelectasis. Second, the robotized system reduced the workload for health care workers (nurses or physiotherapists) and reduced the use and cost of disposable personal protective equipment. Third, passive mobilization of the major joints and muscles reduced pressure sores, venous thromboembolism, and muscle wasting.

#### Robots With Other Potential Roles in Critical Care

In patient evaluation, a KINARM robot (BKIN Technologies Ltd) [48-50] assessed the neurological outcomes of patients. In patients of postcardiac arrest, it accurately and precisely quantified neurological recovery, unlike conventional 5-point rating scales [48]. Similarly, it was able to better quantify neurocognitive impairment in terms of attention, executive function, and visuomotor function in patients of acute kidney injury [49] and patients of post-ICU discharge [50] as compared with Repeatable Battery for the Assessment of Neuropsychological Status, the standardized clinical assessment.

Mechanical compression [51] for cardiopulmonary resuscitation (CPR) using devices such as LUCAS II (Jolife AB) and Corpuls CPR (GS Elektromedizinische Geräte G Stemple GmbH) has been suggested. Although previous RCTs found that such mechanical devices have no clear advantage over manual CPR [52], we believe that mechanical compression technology can be included in future robots to enhance their capabilities. One such trial explored the use of robotic signal–guided CPR [53] to improve survival outcomes. Although no clear advantage of robots was found, this does not preclude further modifications and improvements to CPR-capable robots.

McSleepy [54] is an automated anesthesia drug delivery system for surgery. McSleepy administered appropriate drug doses by monitoring a patient’s level of pain, muscle movements, and depth of consciousness. Although, as of yet, this has only been used in surgery, a closed-loop drug delivery system has tremendous potential for use in critical care settings, which requires constant and precise care.

An intelligent robotic hospital bed, Flexbed [55], with autonomous navigation ability, has been developed for the fast and safe transportation of patients of critical neurosurgery without needing to change beds. Preliminary trials in a simulated crowded hospital corridor environment showed its ability to transport patients quickly, safely, and efficiently while avoiding obstacles with a collision avoidance strategy.

Other robotic applications have been demonstrated in medical training, addressing a broad range of contexts and needs. In pediatric care, a robotic simulator of premature neonates’ wrists [56] was used to train novice caregivers to apply appropriate pressure, eliciting benefits in bone and muscle growth. The WKA-1R robot [57] is an airway simulator that accurately gauges the quality of intubation performance by providing a quantitative and objective determination.

Although this paper focused on patients who are critically ill, there are other potential ways in which robot use can be extended to critical care settings. In gait rehabilitation post spinal cord injury, a robot suit, Hybrid Assistive Limb (Cyberdyne Inc) [58] aided in recovering motor function and gait ability without increasing spasticity in individuals who are paraplegic and nonambulatory. Another robot, the Automatic Recovery Arm Motility Integrated System robot, is a dual exoskeleton robot designed specifically to help with paretic upper limb rehabilitation after stroke [59].

#### Future Research

Although many studies have uncovered the knowledge, attitudes, and perceptions of telepresence robots among health care staff, there were relatively few studies in this area that were conducted on patients themselves. Specifically, in the areas of patient confidentiality and privacy concerns, it might be pertinent to conduct more in-depth studies to uncover patient perspectives with regard to these issues.

In addition, all the included papers only compared the use of robots within the critical care setting before and after their implementation. To get a better idea of the extent to which robots specifically benefit critical care settings, more studies could be done directly comparing the use of robots within critical care versus noncritical care settings; for example, the cost or time benefit of a robot used within the ICU compared with the robot’s use in a normal hospital ward.

As mentioned above, robots are moving toward being able to handle soft skills such as providing comfort. Other than Calmer, there are similar robots currently used outside the ICU, such as Paro [60]. Further development of such robots would be beneficial, especially given that patients in the ICU are more ill and isolated and might require more psychological support.

Currently, little regulation and few protocols exist for the use of robots, despite telepresence having existed for more than a decade. A paper by Clark et al [61] highlighted the types of cyberattacks on robots and the lack of current literature on the economic analysis of cyberattacks on robots. In another study, a protocol was created for the use of Pudu [30], which carefully considered the appropriate and practical use of Pudu in mental health care for isolated patients with COVID-19. It included aspects such as practical frameworks on patient interaction and robot movement, ethical and legal aspects of telecare, and cleaning and disinfection procedures. Although this protocol was newly drawn up for Pudu, it highlights and paves the way for similar protocols and frameworks for worldwide telepresence use. We hope that with more research in this area, suitable regulations and protocols can be implemented to address implementation issues such as manpower replacement in health care, cybersecurity issues, and subsequent ethical and legal consequences.

The papers we found did not mention robots as physician assistants. A physician assistant can accompany a physician to aid in decision-making, diagnosis and interpretation of signs, investigations, or management. Systems that incorporate both AI and robots within the critical setting could allow robots to act as physician assistants. For example, existing AI technology used for the early detection of sepsis [62] can potentially be incorporated into robots. Outside of medicine, many commercial companies are already moving toward incorporating AI into robots. Tesla Inc recently announced its intention to create a humanoid robot that could be used to replace *dangerous, repetitive, boring* tasks [63]. Although much progress is still to be made, it sets a bold tone for future robots that could also be extended to the medical field.

### Limitations of This Review

Our review had several limitations. First, the aim of this project was to identify the different types of robots currently available for use in critical care settings. Currently, there are no theoretical frameworks to classify the types of robots; hence, we categorized the identified robots according to their functionality. However, as the types of robots varied widely, even within each theme, our review could only cover various types of robots in greater breadth rather than depth. In the future, with a larger volume of data, further research could perform detailed comparisons within each functional theme.

Second, we did not include conference abstracts and gray literature in our results as we felt that they did not have sufficient information for us to truly review their benefits and limitations. In addition, the papers were sourced from only 4 databases, which we decided to be the most relevant for the project.

Third, there were limitations in the studies themselves. For example, most studies lacked detailed economic analyses. Parameters such as cost savings, ICU occupancy, and staffing hours were dependent on the existing unique factors and circumstances within each ICU. There were also differences across studies in terms of what was included or omitted during these cost-benefit analyses. For instance, the components that went into calculating cost savings differed: some papers included robot maintenance fees in the overall value, but some did not. Therefore, we were unable to present a generalizable model that could be extrapolated to predict the amount of benefit for all ICUs.

### Conclusions

Robotic use in critical care settings has been rising over the years. In particular, with the current COVID-19 pandemic, there has been greater emphasis on robot use in the ICU, as it allows efficient, safe, and quality contactless care. Although we initially set out believing that robots are more inclined toward aiding in mundane, repetitive tasks, we have discovered that they are capable of delivering substantial value in other more complicated aspects of patient care, including providing superior patient evaluation and rehabilitation. It was interesting to discover robotic use in addressing the softer aspects of patient care through examples such as the Pudu robot and Calmer, which have both proven to be well-integrated and positively perceived in their roles.

However, there are certain barriers that exist to robotic implementation in ICUs. We also hope that our paper will prompt the development of medicolegal frameworks for robotic use, especially in terms of sensitive aspects of care such as patient privacy or medical errors, and in other areas regarding the impact of robotic use, such as job employment.

Overall, given the present roles of robots and many other promising applications, we believe that there is a great opportunity for the further development of robotic technology for critical care, either alone or in combination with AI. If technical, financial, ethical, and legislative barriers to robotic use can be overcome, it would only be a matter of time before robotic presence in critical care becomes ubiquitous.

## Appendix

Multimedia Appendix 1 Search strategies.

## Abbreviations

AI: artificial intelligence
CPR: cardiopulmonary resuscitation
FAST: focused assessment with sonography for trauma
ICU: intensive care unit
NICU: neonatal intensive care unit
PRISMA: Preferred Reporting Items for Systematic Reviews and Meta-Analyses
PROSPERO: International Prospective Register of Systematic Reviews
RCT: randomized controlled trial
TCD: transcranial Doppler
