# Supplementary material for: Use of Robots in Critical Care: Systematic Review
Source: J Med Internet Res. 2022 May 16;24(5):e33380. doi: 10.2196/33380 (PMC9152725; doi:10.2196/33380)
Supplement: Multimedia Appendix 1 [file jmir_v24i5e33380_app1.docx]

**Supplementary File**

Manuscript Title: Use of Robots in Critical Care: A Systematic Review

Authors: Rachel TENG Siew Feng, Yichen DING, Kay Choong SEE

**Tables S1-S3. Search Strategy**

**Table S1. PubMed Search Strategy**

| **Type** | **Terms** |
| --- | --- |
| Robotic | "Robot*” [MeSH Terms] OR “Robot*” [All Fields] NOT “Robot*”[Author Field] |
| Critical care | ICU  “Intensive Care Units”[MeSH Terms] OR “Intensive Care Unit”[All Fields] OR “Intensive Care Units”[All Fields] OR “ICU”[All Fields]  Synonyms of ICU  “Intensive Treatment Unit”[All Fields] OR “Intensive Treatment Units”[All Fields] OR   “Intensive Therapy Unit”[All Fields] OR “Intensive Therapy Units”[All Fields] OR “Intensive Unit Care”[All Fields] OR “Intensive Care”[All Fields] OR “Intensive Unit”[All Fields] OR “Intensive Units”[All Fields]  Subsets of ICU  “Burn Unit”[All Fields] OR “Burn Units”[All Fields] OR “Coronary Care Unit”[All Fields] OR “Coronary Care Units”[All Fields] OR “Respiratory Care Units”[All Fields] OR “Respiratory Care Unit”[All Fields] OR “Intensive Neonatal Care”[All Fields] OR “Intensive Neonatal Unit”[All Fields] OR “Intensive Pediatric Unit”[All Fields] OR “Intensive Pediatric Care”[All Fields] OR “Intensive Paediatric Unit”[All Fields] OR “Intensive Paediatric Care”[All Fields]  HDU  “High Dependency Unit” [All Fields] OR “High Dependency Units” [All Fields] OR “HDU”[All Fields]  Synonyms of HDU  “High Dependency Ward” [All Fields] OR “High Dependency Wards” [All Fields]  Critical Care  “Critical Care”[MeSH Terms] OR “Critical Care”[All Fields] OR “Critical Care Unit”[All Fields] OR “Critical Care Units”[All Fields]  Neonatal Critical Care  “Critical Neonatal Care”[All Fields] OR “Critical Neonatal Unit”[All Fields] OR “Critical Pediatric Care”[All Fields] OR “Critical Pediatric Unit”[All Fields] OR “Critical Paediatric Care”[All Fields] OR “Critical Paediatric Unit”[All Fields] |
| Additional | Intubation  “intubation”[MeSH Terms] OR “intubation”[All Fields] OR “intubations”[All Fields] OR  “Laryngeal Mask”[All Fields] OR “Mask, Laryngeal”[All Fields] OR “Masks, Laryngeal” [All Fields] OR “Laryngeal Mask Airway”[All Fields] OR “Airway, Laryngeal Mask”[All Fields] OR “Airways, Laryngeal Mask”[All Fields] OR “Laryngeal Mask Airways”[All Fields] OR “Rapid Sequence Intubation”[All Fields] OR “Intubation, Rapid Sequence”[All Fields] OR “Rapid Sequence Induction”[All Fields]  Ventilation  “Respiration, artificial”[MesH Terms] OR “artificial respiration”[All Fields] OR “Respiration, artificial”[All Fields] OR “Assisted ventilation”[All Fields] OR “ventilation, Assisted”[All Fields] OR “mechanical ventilation”[All Fields] OR “ventilation, mechanical”[All fields] OR “Artificial ventilation”[All fields] OR “ventilation, Artificial” [All Fields] OR “manual ventilation”[All Fields] OR “ventilation, manual” [All Fields] OR “High-Frequency Ventilation”[All Fields] OR “High-Frequency Oscillation Ventilations”[All Fields] OR “High-Frequency Positive Pressure Ventilation”[All Fields] OR “High-Frequency Jet Ventilation”[All Fields] OR “Jet ventilation”[All Fields] OR “Interactive Ventilatory Support”[All Fields] OR “Neurally Adjusted Ventilatory Assist” [All Fields] OR “Proportional Assist Ventilation”[All Fields] OR “Liquid Ventilation”[All Fields] OR “Fluorocarbon Ventilation”[All Fields] OR “Noninvasive Ventilation”[All Fields] OR “Non-invasive Ventilation”[All Fields] OR “One-Lung Ventilation”[All Fields] OR “One Lung Ventilation”[All Fields] OR “Single-Lung Ventilation”[All Fields] OR “Two-Lung Ventilation”[All Fields] OR “Two Lung Ventilation”[All Fields] OR “Lung Separation Technique”[All Fields] OR “Positive-Pressure Respiration”[All Fields] OR “Positive Pressure Respiration”[All Fields] OR “Positive End-Expiratory Pressure”[All Fields] OR “Continuous Positive Airway Pressure”[All Fields] OR “Continuous Positive Airway Pressure Ventilation”[All Fields] OR “Bilevel Continuous Positive Airway Pressure”[All Fields] OR “Nasal Continuous Positive Airway Pressure”[All Fields] OR “nCPAP Ventilation”[All Fields] OR “CPAP ventilation”[All Fields] OR “Airway Pressure Release Ventilation”[All Fields] OR “APRV Ventilation Mode”[All Fields] OR “Intermittent Positive-Pressure Breathing”[All Fields] OR “Inspiratory Positive-Pressure Breathing”[All Fields] OR “Intermittent Positive-Pressure Ventilation”[All Fields] OR “Biphasic Intermittent Positive Airway Pressure”[All Fields] OR “Inspiratory Positive-Pressure Ventilation”[All Fields] OR “capnometry”[All Fields] OR “high frequency ventilation”[All Fields] OR “hyperinflation”[All Fields] OR “intermittent mandatory ventilation”[All Fields] OR “invasive ventilation”[All Fields] OR “inverse ratio ventilation”[All Fields] OR “negative pressure ventilation”[All Fields] OR “negative-pressure ventilation”[All Fields] OR “pressure controlled ventilation”[All Fields] OR “pressure support ventilation”[All fields] OR “protective ventilation”[All Fields] OR “protective lung ventilation”[All Fields] OR “Ventilator Weaning”[All Fields]  Tracheostomy  “Tracheostomy”[MeSH Terms] OR “Tracheostomy”[All Fields] OR “Tracheostomies”[All Fields] OR “Tracheotomy”[MeSH Terms] OR “Tracheotomy”[All Fields] OR “Tracheotomies”[All Fields] OR “Cricothyroidotomy” [All Fields] OR “Cricothyrotomy”[All Fields]  Cannulation  “Catheterization”[MeSH Terms] OR “Cannulation”[All Fields] OR “Catheterization”[All Fields] OR “Catheterisation”[All Fields] OR “Cannulations” [All Fields] OR “Catheterizations”[All Fields] OR “Catheterisations”[All Fields]  Resuscitation  “resuscitation”[MeSH Terms] OR “resuscitation”[All Fields] OR “resuscitations”[All Fields] OR “CPR”[All Fields] OR “Code Blue”[All Fields] OR “Basic Cardiac Life Support”[All Fields] OR “Advanced Cardiac Life Support”[All Fields] OR “heart massage”[All Fields] OR “Cardiac Massage”[All Fields]  Dialysis  “Dialysis”[All Fields] OR “Dialyses”[All Fields] OR “Renal Dialysis”[MeSH Terms] OR “Hemodialysis”[All Fields] OR “Haemodialysis”[All Fields] OR “Kidneys, Artificial”[MeSH Terms] OR “Artificial Kidneys”[All Fields] OR “Kidney, Artificial”[All Fields] OR “hemodialyse”[All Fields] OR “hemorenodialysis”[All Fields] |
| Combined | ("Robot*” [MeSH Terms] OR “Robot*” [All Fields]) AND (“Intensive Care Units”[MeSH Terms] OR “Intensive Care Unit”[All Fields] OR “Intensive Care Units”[All Fields] OR “ICU”[All Fields] OR “Intensive Treatment Unit”[All Fields] OR “Intensive Treatment Units”[All Fields] OR “Intensive Therapy Unit”[All Fields] OR “Intensive Therapy Units”[All Fields] OR “Intensive Unit Care”[All Fields] OR “Intensive Care”[All Fields] OR “Intensive Unit”[All Fields] OR “Intensive Units”[All Fields] OR “Burn Unit”[All Fields] OR “Burn Units”[All Fields] OR “Coronary Care Unit”[All Fields] OR “Coronary Care Units”[All Fields] OR “Respiratory Care Units”[All Fields] OR “Respiratory Care Unit”[All Fields] OR “Intensive Neonatal Care”[All Fields] OR “Intensive Neonatal Unit”[All Fields] OR “Intensive Pediatric Unit”[All Fields] OR “Intensive Pediatric Care”[All Fields] OR “Intensive Paediatric Unit”[All Fields] OR “Intensive Paediatric Care”[All Fields] OR “High Dependency Unit” [All Fields] OR “High Dependency Units” [All Fields] OR “HDU”[All Fields] OR “High Dependency Ward” [All Fields] OR “High Dependency Wards” [All Fields] OR “Critical Care”[MeSH Terms] OR “Critical Care”[All Fields] OR “Critical Care Unit”[All Fields] OR “Critical Care Units”[All Fields] OR “Critical Neonatal Care”[All Fields] OR “Critical Neonatal Unit”[All Fields] OR “Critical Pediatric Care”[All Fields] OR “Critical Pediatric Unit”[All Fields] OR “Critical Paediatric Care”[All Fields] OR “Critical Paediatric Unit”[All Fields] OR “intubation”[MeSH Terms] OR “intubation”[All Fields] OR “intubations”[All Fields] OR “Laryngeal Mask”[All Fields] OR “Mask, Laryngeal”[All Fields] OR “Masks, Laryngeal” [All Fields] OR “Laryngeal Mask Airway”[All Fields] OR “Airway, Laryngeal Mask”[All Fields] OR “Airways, Laryngeal Mask”[All Fields] OR “Laryngeal Mask Airways”[All Fields] OR “Rapid Sequence Intubation”[All Fields] OR “Intubation, Rapid Sequence”[All Fields] OR “Rapid Sequence Induction”[All Fields] OR “Respiration, artificial”[MesH Terms] OR “artificial respiration”[All Fields] OR “Respiration, artificial”[All Fields] OR “Assisted ventilation”[All Fields] OR “ventilation, Assisted”[All Fields] OR “mechanical ventilation”[All Fields] OR “ventilation, mechanical”[All fields] OR “Artificial ventilation”[All fields] OR “ventilation, Artificial” [All Fields] OR “manual ventilation”[All Fields] OR “ventilation, manual” [All Fields] OR “High-Frequency Ventilation”[All Fields] OR “High-Frequency Oscillation Ventilations”[All Fields] OR “High-Frequency Positive Pressure Ventilation”[All Fields] OR “High-Frequency Jet Ventilation”[All Fields] OR “Jet ventilation”[All Fields] OR “Interactive Ventilatory Support”[All Fields] OR “Neurally Adjusted Ventilatory Assist” [All Fields] OR “Proportional Assist Ventilation”[All Fields] OR “Liquid Ventilation”[All Fields] OR “Fluorocarbon Ventilation”[All Fields] OR “Noninvasive Ventilation”[All Fields] OR “Non-invasive Ventilation”[All Fields] OR “One-Lung Ventilation”[All Fields] OR “One Lung Ventilation”[All Fields] OR “Single-Lung Ventilation”[All Fields] OR “Two-Lung Ventilation”[All Fields] OR “Two Lung Ventilation”[All Fields] OR “Lung Separation Technique”[All Fields] OR “Positive-Pressure Respiration”[All Fields] OR “Positive Pressure Respiration”[All Fields] OR “Positive End-Expiratory Pressure”[All Fields] OR “Continuous Positive Airway Pressure”[All Fields] OR “Continuous Positive Airway Pressure Ventilation”[All Fields] OR “Bilevel Continuous Positive Airway Pressure”[All Fields] OR “Nasal Continuous Positive Airway Pressure”[All Fields] OR “nCPAP Ventilation”[All Fields] OR “CPAP ventilation”[All Fields] OR “Airway Pressure Release Ventilation”[All Fields] OR “APRV Ventilation Mode”[All Fields] OR “Intermittent Positive-Pressure Breathing”[All Fields] OR “Inspiratory Positive-Pressure Breathing”[All Fields] OR “Intermittent Positive-Pressure Ventilation”[All Fields] OR “Biphasic Intermittent Positive Airway Pressure”[All Fields] OR “Inspiratory Positive-Pressure Ventilation”[All Fields] OR “capnometry”[All Fields] OR “high frequency ventilation”[All Fields] OR “hyperinflation”[All Fields] OR “intermittent mandatory ventilation”[All Fields] OR “invasive ventilation”[All Fields] OR “inverse ratio ventilation”[All Fields] OR “negative pressure ventilation”[All Fields] OR “negative-pressure ventilation”[All Fields] OR “pressure controlled ventilation”[All Fields] OR “pressure support ventilation”[All fields] OR “protective ventilation”[All Fields] OR “protective lung ventilation”[All Fields] OR “Ventilator Weaning”[All Fields] OR “Tracheostomy”[MeSH Terms] OR “Tracheostomy”[All Fields] OR “Tracheostomies”[All Fields] OR “Tracheotomy”[MeSH Terms] OR “Tracheotomy”[All Fields] OR “Tracheotomies”[All Fields] OR “Cricothyroidotomy” [All Fields] OR “Cricothyrotomy”[All Fields] OR “Catheterization”[MeSH Terms] OR “Cannulation”[All Fields] OR “Catheterization”[All Fields] OR “Catheterisation”[All Fields] OR “Cannulations” [All Fields] OR “Catheterizations”[All Fields] OR “Catheterisations”[All Fields] OR “resuscitation”[MeSH Terms] OR “resuscitation”[All Fields] OR “resuscitations”[All Fields] OR “CPR”[All Fields] OR “Code Blue”[All Fields] OR “Basic Cardiac Life Support”[All Fields] OR “Advanced Cardiac Life Support”[All Fields] OR “heart massage”[All Fields] OR “Cardiac Massage”[All Fields] OR “Dialysis”[All Fields] OR “Dialyses”[All Fields] OR “Renal Dialysis”[MeSH Terms] OR “Hemodialysis”[All Fields] OR “Haemodialysis”[All Fields] OR “Kidneys, Artificial”[MeSH Terms] OR “Artificial Kidneys”[All Fields] OR “Kidney, Artificial”[All Fields] OR “hemodialyse”[All Fields] OR “hemorenodialysis”[All Fields])  NOT “laparoscopic”[All Fields] |

**Table S2. Embase Search Strategy**

| **Type** | **Terms** |
| --- | --- |
| Robot | “Robotics”/exp OR “Robot*”:ab,ti |
| Critical care | ICU  “Intensive Care”/exp OR “Intensive Care Unit”/exp OR “Intensive Care Unit”:ab,ti OR “Intensive Care Units”:ab,ti OR “ICU”:ab,ti  Synonyms of ICU  “Intensive Treatment Unit”:ab,ti OR “Intensive Treatment Units”:ab,ti OR   “Intensive Therapy Unit”:ab,ti OR “Intensive Therapy Units”:ab,ti OR “Intensive Care Medicine”:ab,ti OR  “Intensive Unit Care”:ab,ti OR “Intensive Care”:ab,ti OR “Intensive Unit”:ab,ti OR “Intensive Units”:ab,ti  Subsets of ICU  “Burn Unit”:ab,ti OR “Burn Units”:ab,ti OR “Coronary Care Unit”:ab,ti OR “Coronary Care Units”:ab,ti OR “Respiratory Care Units”:ab,ti OR “Respiratory Care Unit”:ab,ti OR “Intensive Neonatal Care”:ab,ti OR “Intensive Neonatal Unit”:ab,ti OR “Intensive Pediatric Unit”:ab,ti OR “Intensive Pediatric Care”:ab,ti OR “Intensive Paediatric Unit”:ab,ti OR “Intensive Paediatric Care”:ab,ti  Synonyms of HDU  “High Dependency Unit”/exp OR “High Dependency Unit”:ab,ti OR “High Dependency Units”:ab,ti OR “High Dependancy Unit”:ab,ti OR “High Dependancy Units”:ab,ti OR “HDU”:ab,ti  OR “High Dependency Ward”:ab,ti OR “High Dependency Wards”:ab,ti  Critical Care  “Critical Care”:ab,ti OR “Critical Care Unit”:ab,ti OR “Critical Care Units”:ab,ti  Neonatal Critical Care  “Critical Neonatal Care”:ab,ti OR “Critical Neonatal Unit”:ab,ti OR “Critical Pediatric Care”:ab,ti OR “Critical Pediatric Unit”:ab,ti OR “Critical Paediatric Care”:ab,ti OR “Critical Paediatric Unit”:ab,ti |
| Additional | Intubation  “intubation”/exp OR “intubation”:ab,ti OR “intubations”:ab,ti  “Laryngeal Mask”:ab,ti OR “Mask, Laryngeal”:ab,ti OR “Masks, Laryngeal”:ab,ti OR “Laryngeal Mask Airway”:ab,ti OR “Airway, Laryngeal Mask”:ab,ti OR “Airways, Laryngeal Mask”:ab,ti OR “Laryngeal Mask Airways”:ab,ti OR “Rapid Sequence Intubation”:ab,ti OR “Intubation, Rapid Sequence”:ab,ti OR “Rapid Sequence Induction”:ab,ti  Ventilation  “Artificial ventilation”/exp OR “Artificial ventilation”:ab,ti OR “Ventilation, Artificial”:ab,ti OR “artificial respiration”:ab,ti OR “Respiration, artificial”:ab,ti OR “Mechanical ventilation”:ab,ti OR “Ventilation, Mechanical”:ab,ti OR “Assisted ventilation”:ab,ti OR “Ventilation, Assisted”:ab,ti OR “manual ventilation”:ab,ti OR “Ventilation, Manual”:ab,ti OR “High-Frequency Ventilation”:ab,ti OR “High-Frequency Oscillation Ventilations”:ab,ti OR “High-Frequency Positive Pressure Ventilation”:ab,ti OR “High-Frequency Jet Ventilation”:ab,ti OR “Jet ventilation”:ab,ti OR “Interactive Ventilatory Support”:ab,ti OR “Neurally Adjusted Ventilatory Assist”:ab,ti OR “Proportional Assist Ventilation”:ab,ti OR “Liquid Ventilation”:ab,ti OR “Fluorocarbon Ventilation”:ab,ti OR “Noninvasive Ventilation”:ab,ti OR “Non-invasive Ventilation”:ab,ti OR “One-Lung Ventilation”:ab,ti OR “One Lung Ventilation”:ab,ti OR “Single-Lung Ventilation”:ab,ti OR “Two-Lung Ventilation”:ab,ti OR “Two Lung Ventilation”:ab,ti OR “Lung Separation Technique”:ab,ti OR “Positive-Pressure Respiration”:ab,ti OR “Positive Pressure Respiration”:ab,ti OR “Positive End-Expiratory Pressure”:ab,ti OR “Continuous Positive Airway Pressure”:ab,ti OR “Continuous Positive Airway Pressure Ventilation”:ab,ti OR “Bilevel Continuous Positive Airway Pressure”:ab,ti OR “Nasal Continuous Positive Airway Pressure”:ab,ti OR “nCPAP Ventilation”:ab,ti OR “CPAP ventilation”:ab,ti OR “Airway Pressure Release Ventilation”:ab,ti OR “APRV Ventilation Mode”:ab,ti OR “Intermittent Positive-Pressure Breathing”:ab,ti OR “Inspiratory Positive-Pressure Breathing”:ab,ti OR “Intermittent Positive-Pressure Ventilation”:ab,ti OR “Biphasic Intermittent Positive Airway Pressure”:ab,ti OR “Inspiratory Positive-Pressure Ventilation”:ab,ti OR “capnometry”:ab,ti OR “high frequency ventilation”:ab,ti OR “hyperinflation”:ab,ti OR “intermittent mandatory ventilation”:ab,ti OR “invasive ventilation”:ab,ti OR “inverse ratio ventilation”:ab,ti OR “negative pressure ventilation”:ab,ti OR “negative-pressure ventilation”:ab,ti OR “pressure controlled ventilation”:ab,ti OR “pressure support ventilation”:ab,ti OR “protective ventilation”:ab,ti OR “protective lung ventilation”:ab,ti OR “Ventilator Weaning”:ab,ti  Tracheostomy  “Tracheostomy”/exp OR “Tracheostomy”:ab,ti OR “Tracheostomies”:ab,ti OR “Tracheotomy”/exp OR “Tracheotomy”:ab,ti OR “Tracheotomies”:ab,ti OR “Cricothyroidotomy”:ab,ti OR “Cricothyrotomy”:ab,ti  Cannulation  “Cannulation”/exp OR “Catheterization”/exp OR “Cannulation”:ab,ti OR “Catheterization”:ab,ti OR “Catheterisation”:ab,ti OR “Cannulations”:ab,ti OR “Catheterizations”:ab,ti OR “Catheterisations”:ab,ti  Resuscitation  “resuscitation”/exp OR “resuscitation”:ab,ti OR “resuscitations”:ab,ti OR “CPR”:ab,ti OR “Code Blue”:ab,ti OR “Basic Cardiac Life Support”:ab,ti OR “Advanced Cardiac Life Support”:ab,ti OR “heart massage”:ab,ti OR “Cardiac Massage”:ab,ti  Dialysis  “Dialysis”/exp OR “Dialysis”:ab,ti OR “Dialyses”:ab,ti OR “Hemodialysis”:ab,ti OR “Haemodialysis”:ab,ti OR “Kidneys, Artificial”:ab,ti OR “Artificial Kidneys”:ab,ti OR “Kidney, Artificial”:ab,ti OR “extracorporeal blood cleansing”:ab,ti OR “hemodialyse”:ab,ti OR “hemorenodialysis”:ab,ti |
| Combined | (“Robotics”/exp OR “Robot*”:ab,ti) AND (“Intensive Care”/exp OR “Intensive Care Unit”/exp OR “Intensive Care Unit”:ab,ti OR “Intensive Care Units”:ab,ti OR “ICU”:ab,ti OR “Intensive Treatment Unit”:ab,ti OR “Intensive Treatment Units”:ab,ti OR “Intensive Therapy Unit”:ab,ti OR “Intensive Therapy Units”:ab,ti OR “Intensive Care Medicine”:ab,ti OR “Intensive Unit Care”:ab,ti OR “Intensive Care”:ab,ti OR “Intensive Unit”:ab,ti OR “Intensive Units”:ab,ti OR “Burn Unit”:ab,ti OR “Burn Units”:ab,ti OR “Coronary Care Unit”:ab,ti OR “Coronary Care Units”:ab,ti OR “Respiratory Care Units”:ab,ti OR “Respiratory Care Unit”:ab,ti OR “Intensive Neonatal Care”:ab,ti OR “Intensive Neonatal Unit”:ab,ti OR “Intensive Pediatric Unit”:ab,ti OR “Intensive Pediatric Care”:ab,ti OR “Intensive Paediatric Unit”:ab,ti OR “Intensive Paediatric Care”:ab,ti OR “High Dependency Unit”/exp OR “High Dependency Unit”:ab,ti OR “High Dependency Units”:ab,ti OR “High Dependancy Unit”:ab,ti OR “High Dependancy Units”:ab,ti OR “HDU”:ab,ti OR “High Dependency Ward”:ab,ti OR “High Dependency Wards”:ab,ti OR “Critical Care”:ab,ti OR “Critical Care Unit”:ab,ti OR “Critical Care Units”:ab,ti OR “Critical Neonatal Care”:ab,ti OR “Critical Neonatal Unit”:ab,ti OR “Critical Pediatric Care”:ab,ti OR “Critical Pediatric Unit”:ab,ti OR “Critical Paediatric Care”:ab,ti OR “Critical Paediatric Unit”:ab,ti OR “intubation”:ab,ti OR “intubations”:ab,ti OR “Laryngeal Mask”:ab,ti OR “Mask, Laryngeal”:ab,ti OR “Masks, Laryngeal”:ab,ti OR “Laryngeal Mask Airway”:ab,ti OR “Airway, Laryngeal Mask”:ab,ti OR “Airways, Laryngeal Mask”:ab,ti OR “Laryngeal Mask Airways”:ab,ti OR “Rapid Sequence Intubation”:ab,ti OR “Intubation, Rapid Sequence”:ab,ti OR “Rapid Sequence Induction”:ab,ti OR “Artificial ventilation”:ab,ti OR “Ventilation, Artificial”:ab,ti OR “artificial respiration”:ab,ti OR “Respiration, artificial”:ab,ti OR “Mechanical ventilation”:ab,ti OR “Ventilation, Mechanical”:ab,ti OR “Assisted ventilation”:ab,ti OR “Ventilation, Assisted”:ab,ti OR “manual ventilation”:ab,ti OR “Ventilation, Manual”:ab,ti OR “High-Frequency Ventilation”:ab,ti OR “High-Frequency Oscillation Ventilations”:ab,ti OR “High-Frequency Positive Pressure Ventilation”:ab,ti OR “High-Frequency Jet Ventilation”:ab,ti OR “Jet ventilation”:ab,ti OR “Interactive Ventilatory Support”:ab,ti OR “Neurally Adjusted Ventilatory Assist”:ab,ti OR “Proportional Assist Ventilation”:ab,ti OR “Liquid Ventilation”:ab,ti OR “Fluorocarbon Ventilation”:ab,ti OR “Noninvasive Ventilation”:ab,ti OR “Non-invasive Ventilation”:ab,ti OR “One-Lung Ventilation”:ab,ti OR “One Lung Ventilation”:ab,ti OR “Single-Lung Ventilation”:ab,ti OR “Two-Lung Ventilation”:ab,ti OR “Two Lung Ventilation”:ab,ti OR “Lung Separation Technique”:ab,ti OR “Positive-Pressure Respiration”:ab,ti OR “Positive Pressure Respiration”:ab,ti OR “Positive End-Expiratory Pressure”:ab,ti OR “Continuous Positive Airway Pressure”:ab,ti OR “Continuous Positive Airway Pressure Ventilation”:ab,ti OR “Bilevel Continuous Positive Airway Pressure”:ab,ti OR “Nasal Continuous Positive Airway Pressure”:ab,ti OR “nCPAP Ventilation”:ab,ti OR “CPAP ventilation”:ab,ti OR “Airway Pressure Release Ventilation”:ab,ti OR “APRV Ventilation Mode”:ab,ti OR “Intermittent Positive-Pressure Breathing”:ab,ti OR “Inspiratory Positive-Pressure Breathing”:ab,ti OR “Intermittent Positive-Pressure Ventilation”:ab,ti OR “Biphasic Intermittent Positive Airway Pressure”:ab,ti OR “Inspiratory Positive-Pressure Ventilation”:ab,ti OR “capnometry”:ab,ti OR “high frequency ventilation”:ab,ti OR “hyperinflation”:ab,ti OR “intermittent mandatory ventilation”:ab,ti OR “invasive ventilation”:ab,ti OR “inverse ratio ventilation”:ab,ti OR “negative pressure ventilation”:ab,ti OR “negative-pressure ventilation”:ab,ti OR “pressure controlled ventilation”:ab,ti OR “pressure support ventilation”:ab,ti OR “protective ventilation”:ab,ti OR “protective lung ventilation”:ab,ti OR “Ventilator Weaning”:ab,ti OR “Tracheostomy”:ab,ti OR “Tracheostomies”:ab,ti OR “Tracheotomy”:ab,ti OR “Tracheotomies”:ab,ti OR “Cricothyroidotomy”:ab,ti OR “Cricothyrotomy”:ab,ti OR “Cannulation”:ab,ti OR “Catheterization”:ab,ti OR “Catheterisation”:ab,ti OR “Cannulations”:ab,ti OR “Catheterizations”:ab,ti OR “Catheterisations”:ab,ti OR “resuscitation”:ab,ti OR “resuscitations”:ab,ti OR “CPR”:ab,ti OR “Code Blue”:ab,ti OR “Basic Cardiac Life Support”:ab,ti OR “Advanced Cardiac Life Support”:ab,ti OR “heart massage”:ab,ti OR “Cardiac Massage”:ab,ti OR “Dialysis”:ab,ti OR “Dialyses”:ab,ti OR “Hemodialysis”:ab,ti OR “Haemodialysis”:ab,ti OR “Kidneys, Artificial”:ab,ti OR “Artificial Kidneys”:ab,ti OR “Kidney, Artificial”:ab,ti OR “extracorporeal blood cleansing”:ab,ti OR “hemodialyse”:ab,ti OR “hemorenodialysis”:ab,ti) NOT “laparoscopic”:ab,ti |

**Table S3. IEEE Xplore and ACM Library Search Strategy**

| Robot | ("Robot*" OR "Robot*") |
| --- | --- |
| Critical Care | ("Intensive Care Unit" OR "Intensive Care Units" OR "ICU" OR "Intensive Treatment Unit" OR "Intensive Treatment Units" OR "Intensive Therapy Unit" OR "Intensive Therapy Units" OR "Intensive Unit Care" OR "Intensive Care" OR "Intensive Unit" OR "Intensive Units" OR "Burn Unit" OR "Burn Units" OR "Coronary Care Unit” OR "Coronary Care Units" OR "Respiratory Care Units" OR "Respiratory Care Unit” OR "Intensive Neonatal Care" OR "Intensive Neonatal Unit" OR "Intensive Pediatric Unit" OR "Intensive Pediatric Care" OR "Intensive Paediatric Unit" OR "Intensive Paediatric Care" OR "High Dependency Unit" OR "High Dependency Units" OR "HDU" OR "High Dependency Ward" OR "High Dependency Wards” OR "Critical Care" OR "Critical Care Unit" OR "Critical Care Units" OR "Critical Neonatal Care" OR "Critical Neonatal Unit" OR "Critical Pediatric Care" OR "Critical Pediatric Unit" OR "Critical Paediatric Care" OR "Critical Paediatric Unit") |
| Combined | ("Robot*" OR "Robot*") AND ("Intensive Care Unit" OR "Intensive Care Units" OR "ICU" OR "Intensive Treatment Unit" OR "Intensive Treatment Units" OR "Intensive Therapy Unit" OR "Intensive Therapy Units" OR "Intensive Unit Care" OR "Intensive Care" OR "Intensive Unit" OR "Intensive Units" OR "Burn Unit" OR "Burn Units" OR "Coronary Care Unit” OR "Coronary Care Units" OR "Respiratory Care Units" OR "Respiratory Care Unit” OR "Intensive Neonatal Care" OR "Intensive Neonatal Unit" OR "Intensive Pediatric Unit" OR "Intensive Pediatric Care" OR "Intensive Paediatric Unit" OR "Intensive Paediatric Care" OR "High Dependency Unit" OR "High Dependency Units" OR "HDU" OR "High Dependency Ward" OR "High Dependency Wards” OR "Critical Care" OR "Critical Care Unit" OR "Critical Care Units" OR "Critical Neonatal Care" OR "Critical Neonatal Unit" OR "Critical Pediatric Care" OR "Critical Pediatric Unit" OR "Critical Paediatric Care" OR "Critical Paediatric Unit") |
